# Curcumin: A multi-target disease-modifying agent for late-stage transthyretin amyloidosis

**DOI:** 10.1038/srep26623

**Published:** 2016-05-20

**Authors:** Nelson Ferreira, Nádia P. Gonçalves, Maria J. Saraiva, Maria R. Almeida

**Affiliations:** 1 IBMC - Instituto de Biologia Molecular e Celular, Universidade do Porto, Rua Alfredo Allen, 208, 4200 – 135 Porto, Portugal; 2 i3S – Instituto de Investigação e Inovação em Saúde da Universidade do Porto, Rua Alfredo Allen, 208, 4200 – 135 Porto, Portugal; 3 ICBAS, Instituto de Ciências Biomédicas Abel Salazar, Universidade do Porto, Rua Jorge Viterbo Ferreira 228, 4050 – 313 Porto, Portugal

## Abstract

Transthyretin amyloidoses encompass a variety of acquired and hereditary diseases triggered by systemic extracellular accumulation of toxic transthyretin aggregates and fibrils, particularly in the peripheral nervous system. Since transthyretin amyloidoses are typically complex progressive disorders, therapeutic approaches aiming multiple molecular targets simultaneously, might improve therapy efficacy and treatment outcome. In this study, we evaluate the protective effect of physiologically achievable doses of curcumin on the cytotoxicity induced by transthyretin oligomers *in vitro* by showing reduction of caspase-3 activity and the levels of endoplasmic reticulum-resident chaperone binding immunoglobulin protein. When given to an aged Familial Amyloidotic Polyneuropathy mouse model, curcumin not only reduced transthyretin aggregates deposition and toxicity in both gastrointestinal tract and dorsal root ganglia but also remodeled congophilic amyloid material in tissues. In addition, curcumin enhanced internalization, intracellular transport and degradation of transthyretin oligomers by primary macrophages from aged Familial Amyloidotic Polyneuropathy transgenic mice, suggesting an impaired activation of naïve phagocytic cells exposed to transthyretin toxic intermediate species. Overall, our results clearly support curcumin or optimized derivatives as promising multi-target disease-modifying agent for late-stage transthyretin amyloidosis.

The accumulation of misfolded proteins as insoluble amyloid fibrils is a key pathognomonic feature of many neurodegenerative diseases, including Familial Amyloidotic Polyneuropathy (FAP), type II diabetes, Alzheimer’s, Parkinson’s and Huntington’s diseases. In most of these pathologies, the end stage aggregated species that accumulate in tissues are composed mostly by 6–10 nm twisted β-pleated-fibrils, in which the polypeptide chain is arranged in sheets perpendicular to the fibril axis being hydrogen bonding parallel^1^. Although amyloid fibrils have been reported to be toxic^2^, accumulating evidence suggests that the non-fibrillar oligomer intermediaries are the primary culprits for neurodegeneration and exert their toxicity through distinct mechanisms^3^.

Transthyretin (TTR) is an abundant plasma protein that is mainly synthesized by the liver and choroid plexuses of the brain. TTR in its homotetrameric structure acts as a carrier protein for thyroid hormones and retinol-binding protein in plasma and cerebrospinal fluid^4^. Extracellular TTR aggregation and deposition triggers inflammation and oxidative damage^5^, disruption of calcium homeostasis^6^, extracellular matrix remodeling^7^, activation of heat shock response and endoplasmic reticulum (ER) stress pathways^6^,^8^, including common molecular actors and scenarios, that resemble in many features those associated to local amyloidoses affecting the central nervous system such as Alzheimer’s and Parkinson’s diseases.

FAP is a fatal neurodegenerative disorder characterized by the extracellular deposition of aggregates and fibrils of mutant forms of TTR, particularly in peripheral nerves and ganglia of the peripheral nervous system. Although the most common TTR mutation leading to FAP gives origin to a substitution of methionine for valine at position 30 (TTR V30M), more than one hundred amyloidogenic TTR variants have been described. Different mutations in the TTR gene are associated with distinct age of onset, disease penetrance, clinical phenotype, prognosis and clinical outcome^9^. In addition to familial TTR- associated forms of amyloid, senile systemic amyloidosis (SSA) is a nonhereditary, late onset sporadic form of TTR amyloidosis that is typically related with wild-type TTR amyloid deposition in the heart, leading to cardiac dysfunction and ultimately death^10^.

Through a combination of compound library screens and epidemiological data, many low molecular weight organic compounds have been suggested as effective therapeutic and prophylactic agents against amyloidosis. The most striking aspect that has emerged from recent studies is that some of these small molecules, in particular natural occurring polyphenols, do not act on a single target in the multi-step amyloidogenic cascade; rather, they seem to inhibit/redirect different steps of the amyloid molecular dynamics and also modulate the biology of cells and tissues where amyloid deposition takes place^11^,^12^. Among those polyphenols, curcumin, a natural occurring diarylheptanoid isolated from Curcuma longa, has been found to modulate oligomerization and fibril formation of several amyloid proteins, including TTR^12^,^13^, α-synuclein^14^ and amyloid-β^15^.

Curcumin has anti-oxidant and anti-inflammatory properties that have also been suggested to play a protective role in a variety of chronic pathologies such as cancer, atherosclerosis, and neurodegenerative diseases^16^. In addition, curcumin can efficiently chelate amyloid associated metal ions, such as Fe^2+^, Zn^2+^ and Cu^2+^ which may reduce amyloid aggregation or oxidative neurotoxicity *in vivo*^17^. Furthermore, curcumin is widely used as therapeutic agent in Chinese and Indian Ayurvedic medicine and is “generally recognized as safe” by the U.S. Food and Drug Administration. Epidemiological studies conducted in India, where curcumin is highly consumed, showed that Alzheimer’s disease incidence is significantly lower than that reported from the western world^18^.

These observations together with the findings that curcumin disaggregates TTR amyloid fibrils *in vitro*^11^ and activates innate immune system promoting amyloid clearance^19^, prompted us to investigate the effect of curcumin supplementation in a late-stage mouse model for FAP.

## Results

### Curcumin reduces ER stress and protects Schwann cells from entering into the apoptotic signaling pathway upon exposure to TTR aggregates

**Figure 1:**
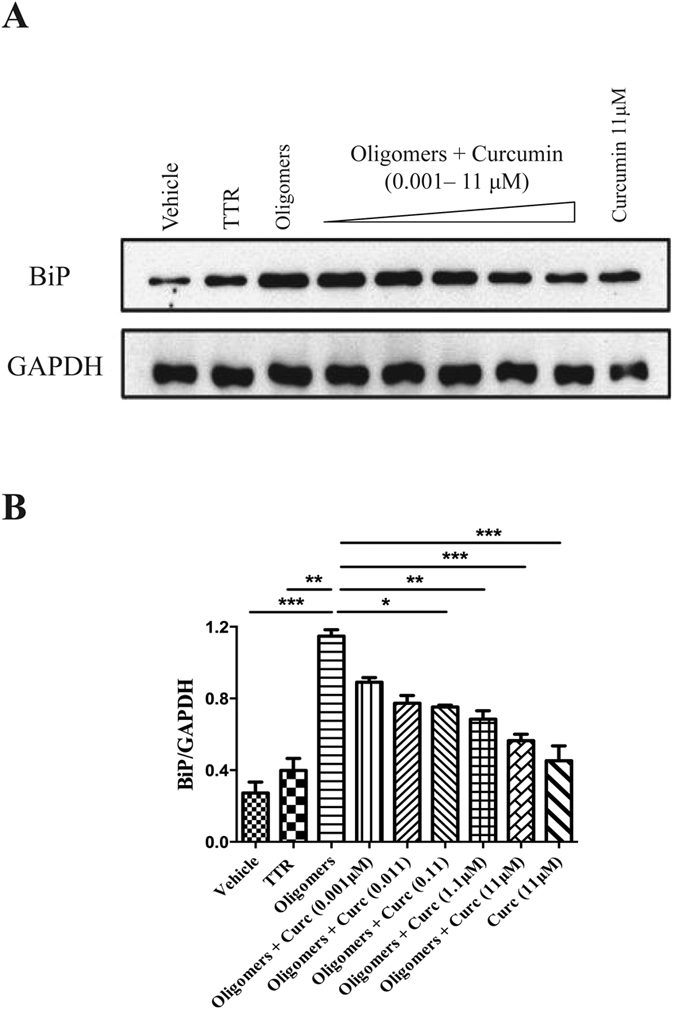
Curcumin inhibits TTR oligomers-dependent activation of ER-chaperone BiP. (**A**) Anti-BiP Western blot of protein extracts from Schwannoma cells exposed to soluble TTR Y78F (TTR) or to TTR Y78F oligomers (Oligomers) or TTR Y78F oligomers pre-treated with curcumin for 24 h (final concentration of protein in cell culture medium 2 μM and curcumin 0.001–11 μM). In parallel, cells were incubated with curcumin alone, at highest concentration used (Curc. 11 μM). (**B**) Bar graph represents quantification of data and is presented as mean ± SEM (*p < 0.05; **p < 0.01; ***p < 0.001).

We investigated the impact of curcumin on TTR aggregate-induced ER stress response and apoptosis by exposing RN22 cells to TTR oligomers formed in the absence or presence of different curcumin concentrations. As expected from previous work^6^, TTR oligomers, but not soluble TTR, triggered a remarkable increase in the intracellular amount of BiP, the central regulator for the ER-stress response^20^, indicating cellular toxicity (Fig. 1). In strong contrast, we observed no such effect when identical amounts of oligomers formed in the presence of curcumin were added to the RN22 cells, indicating that these species are less toxic than untreated oligomeric intermediaries (Fig. 1). Moreover, exposure of cells to curcumin alone (up to 11 μM) did not interfere with BiP expression along the range of concentrations used in the assay.

**Figure 2:**
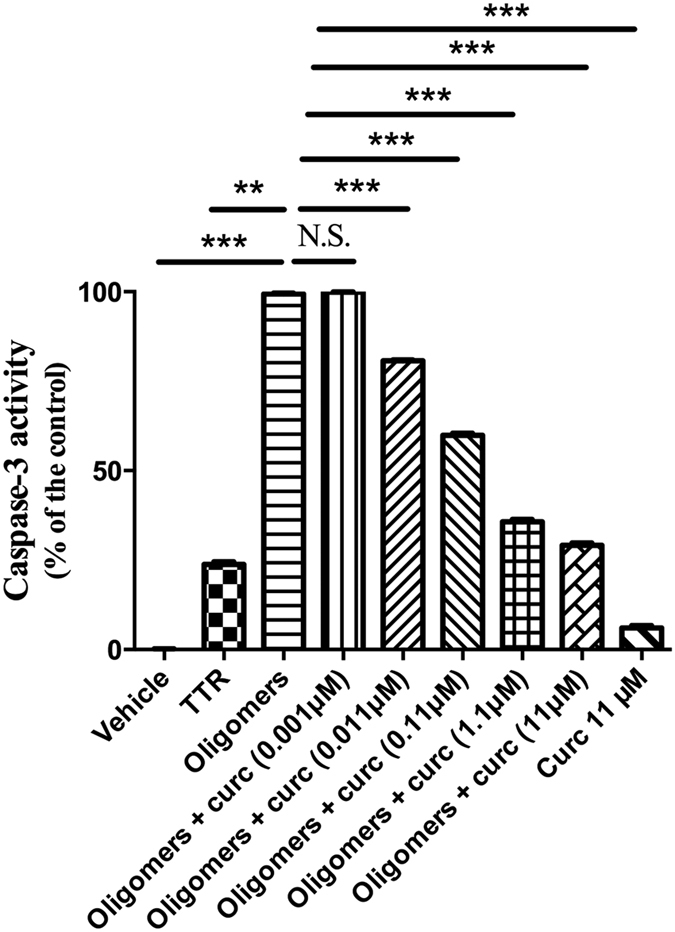
Curcumin reduces caspase-3 activity associated with extracellular TTR oligomers. Activity of caspase-3 was measured in Schwannoma cells exposed to soluble TTR Y78F (TTR) or to TTR Y78F oligomers (Oligomers) or TTR Y78F oligomers pre-treated with curcumin for 24 h (same conditions as indicated in Fig. 1) (**p < 0.01; ***p < 0.001; N.S. = non-significant).

In addition, toxicity of TTR aggregate intermediaries was also studied through a standardized caspase-3 assay in this Schwann cell line. Unlike soluble TTR, TTR oligomeric species induced substantial caspase-3 activity in RN22 cells, indicative of cell damage and entry into the apoptotic signaling pathway. On the other hand, cell exposure to oligomers formed in the presence of curcumin resulted in a significant dose-dependent reduction of caspase-3 activity (Fig. 2). Moreover, no significant alteration in this enzyme activity was noted when cells were incubated with curcumin alone (11 μM) (Fig. 2).

### Evaluation of curcumin effect in a late-stage FAP mouse model

In view of the promising data described above and available in the literature concerning *in vitro* and *in vivo* studies^12^,^13^,^21^, we investigated the effect of dietary supplementation of curcumin on TTR amyloid formation using a late-stage FAP mouse model expressing the amyloidogenic human TTR V30M variant on a *Hsf-1* heterozygous background (hTTR V30M/Hsf)^8^. The impairment of *Hsf-1* expression leads to an extensive and early deposition of non-fibrillar TTR in several tissues, including the gastrointestinal tract and the peripheral nervous system. Non-fibrillar TTR species start to deposit between 3–6 months of age and gradually evolve to congophilic material, typically after 12 months of age. Therefore, this mouse model is highly relevant for testing new therapeutic strategies targeting different stages of the pathology.

In the current study, we aimed at evaluating the effect of curcumin in a late-stage disease in which deposition of non-fibrillar TTR coexists with birefringent congophilic material in tissues. Protocol design, drug dosage and selection of endpoints were based on information from a previous study^13^. As expected, curcumin supplementation was very well-tolerated in aged mice and did not produce any observable adverse side effects. No alteration was observed in body weight or mortality between animals treated with curcumin and age-matched controls. In addition, no histological abnormalities were observed in liver sections stained with hematoxylin and eosin for morphologic assessment (data not shown).

### Curcumin binds to plasma TTR at the thyroxine binding sites and increases its resistance to dissociation

To confirm curcumin binding to TTR *in vivo*, plasma from mice fed with curcumin-enriched diet or standard diet (control) was incubated with radiolabeled thyroxine (T_4_) ([^125^I]-T_4_) and subjected to gel electrophoresis under native conditions. T_4_-binding proteins were visualized by phosphor imaging analysis, as shown in Supp. Fig. 1. The results showed that samples from curcumin treated mice presented considerably less intense TTR bands, indicating that after gut absorption and systemic distribution, curcumin selectively competes with T_4_ for its binding to TTR (41.74% competition) (Supp. Fig. 1). Densitometry analysis of the IEF gels demonstrated that curcumin chronic supplementation significantly increases TTR resistance to dissociation when compared with plasma TTR from untreated animals under the tested conditions (Supp. Fig. 2).

### Curcumin decreases TTR deposition and rescues tissue toxicity

TTR levels in plasma were quantified by radial immunodiffusion and showed no statistical difference between mice fed with curcumin-enriched diet and control mice (556.8 ± 5.1 μg TTR/ml and 556.2 ± 5.5 μg TTR/ml, respectively), indicating that curcumin administration did not affect TTR liver synthesis and turnover *in vivo*.

**Figure 3:**
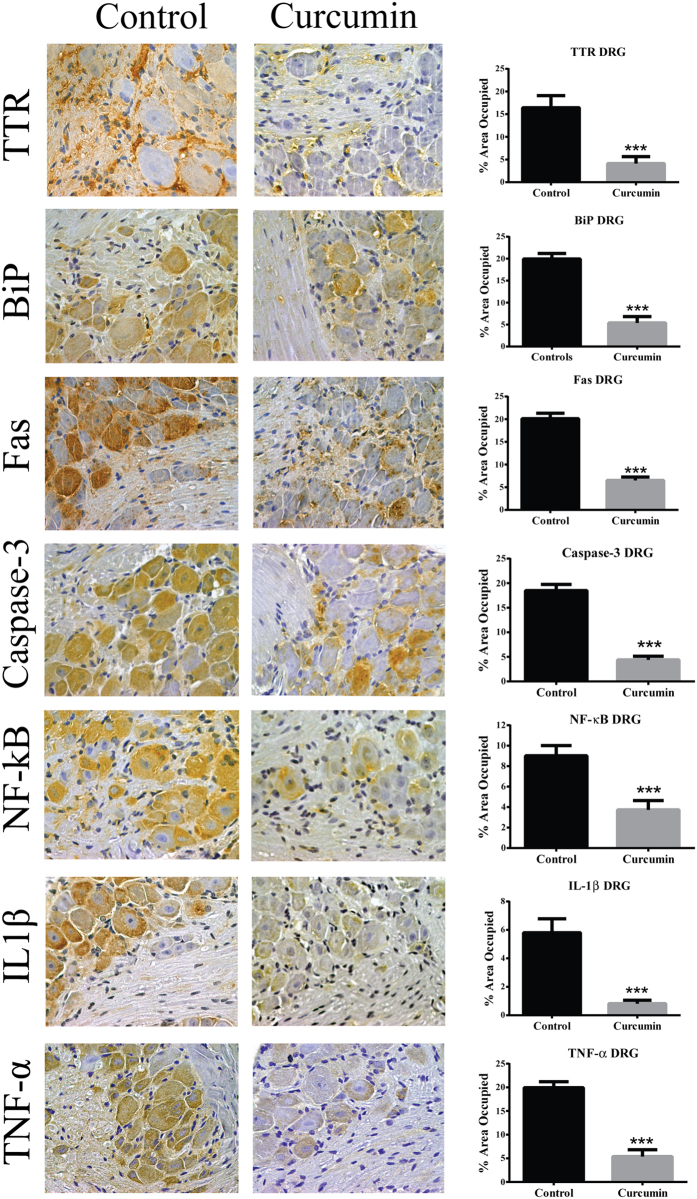
Curcumin decreases TTR deposition and associated toxicity in DRG of aged hTTR V30M/Hsf mice. Representative images of immunohistochemistry analysis of TTR, BiP, Fas death receptor, cleaved caspase-3, NF-κB, IL-1β and TNF-α in DRG of mice treated with curcumin (right panels; *n* = 12) and age-matched controls (left panels; *n* = 14); 40× magnification. Bar graphs: quantification of immunohistochemistry images is represented as percentage of area occupied ± SEM (***p < 0.001). These observations were supported by Western blot analysis of TTR levels after normalization to GAPDH signal intensities (Fig. 4).

**Figure 4:**
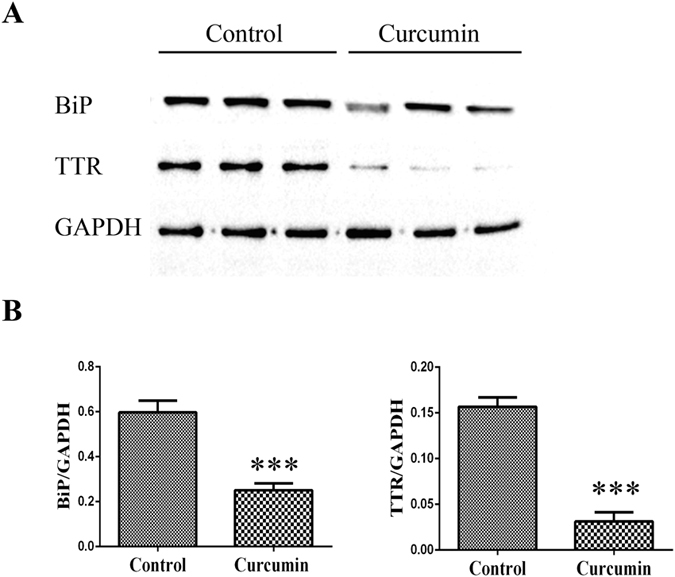
(**A**) Representative anti-BiP and anti-TTR Western blots of DRG from curcumin- treated (*n* = 6) and control mice (*n* = 6). (**B**) Bar graphs illustrate normalized BiP/GAPDH and TTR/GAPDH density quantifications ± SEM (***p < 0.001). Similar results were found throughout the GI tract, in particular in stomach (Figs 5 and 6).

**Figure 5:**
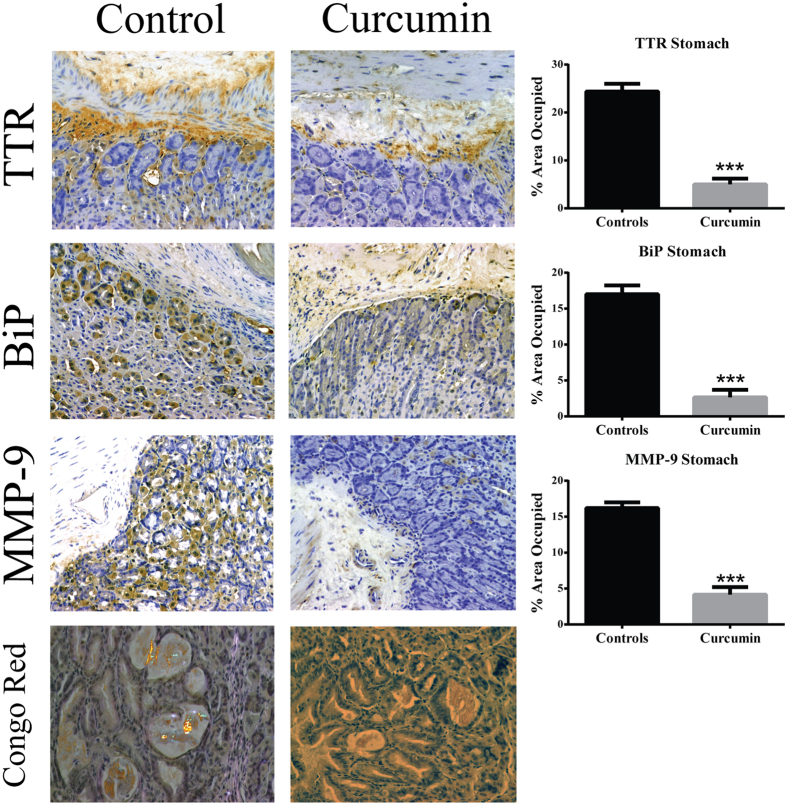
Curcumin decreases TTR aggregates deposition and associated toxicity in the stomach of aged hTTR V30M/Hsf mice. Representative images of immunohistochemistry analysis of TTR, BiP, MMP-9 and also Congo red staining of stomach sections of mice treated with curcumin (right panels; *n* = 12) and age-matched controls (left panels; *n* = 14); 20× magnification. Bar graphs: quantification of immunohistochemistry images is represented as percentage of area occupied ± SEM (***p < 0.001).

**Figure 6:**
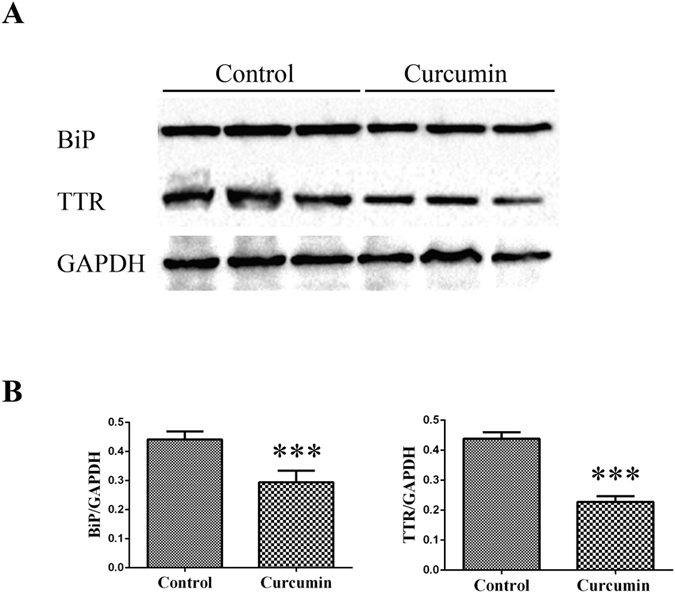
(**A**) Representative anti-BiP and anti-TTR Western blots of stomachs from curcumin-treated (*n* = 6) and control mice (*n* = 6). (**B**) Bar graphs: normalized BiP/GAPDH and TTR/GAPDH density quantifications ± SEM (***p < 0.001).

The effect of curcumin supplementation on TTR deposition and tissue damage was analyzed by SQ-IHC and western blotting in different target organs. At the end of treatment, mice were 15.5 months of age thus, as expected, control animals showed widespread TTR deposition throughout the interstitial connective tissue of DRG, surrounding the perikaryon and in close contact with satellite glial cells^8^. In strong contrast, sub-chronic administration of curcumin inhibited TTR deposition in ganglia associated with spinal nerves, as denoted in Figs 3 and 4. Similar results were found in stomach (Figs 5 and 6).

Since extracellular accumulation of TTR aggregates closely correlates with cellular damage and toxicity^5^,^22^, we next investigated the effect of curcumin treatment on several molecular markers associated with the disease in the peripheral nervous system and gastrointestinal tract. In both DRG (Figs 3 and 4) and stomach (Figs 5 and 6), we found reduced levels of ER-chaperone BiP. In addition, reduced levels of Fas death receptor were observed in curcumin treated mice compared to controls (Fig. 3).

Supporting these observations, several key pro-inflammatory mediators associated with disease pathogenesis, namely NF-κB, IL-1β and TNF-α^8^, were found reduced in DRG of curcumin treated mice (Fig. 3). Overall, these results sustained that curcumin modulates TTR deposition in both gastrointestinal tract and peripheral nervous system.

### Curcumin remodels amyloid deposits and enhances its clearance

Previous data has shown that curcumin disaggregates TTR fibrils *in vitro*^12^. This prompted us to investigate the effect of curcumin in amyloid deposition and extracellular matrix remodeling. The presence of amyloid was detected by Congo red staining in stomach, the primary amyloid target-organ in this particular mouse model.

The results showed that 7 out of 14 (50%) control animals exhibited Congo Red-positive material, whereas only 1 out of 12 (8%) curcumin treated animals presented congophilic amyloid deposits (Fig. 5). These results were corroborated by the reduced levels of MMP-9 (biomarker associated with amyloid extracellular deposition) in stomach (Fig. 5), thus clearly indicating that curcumin remodels TTR amyloid deposits and extracellular matrix *in vivo*.

### Curcumin increases internalization and intracellular transport of TTR aggregates in macrophages

**Figure 7:**
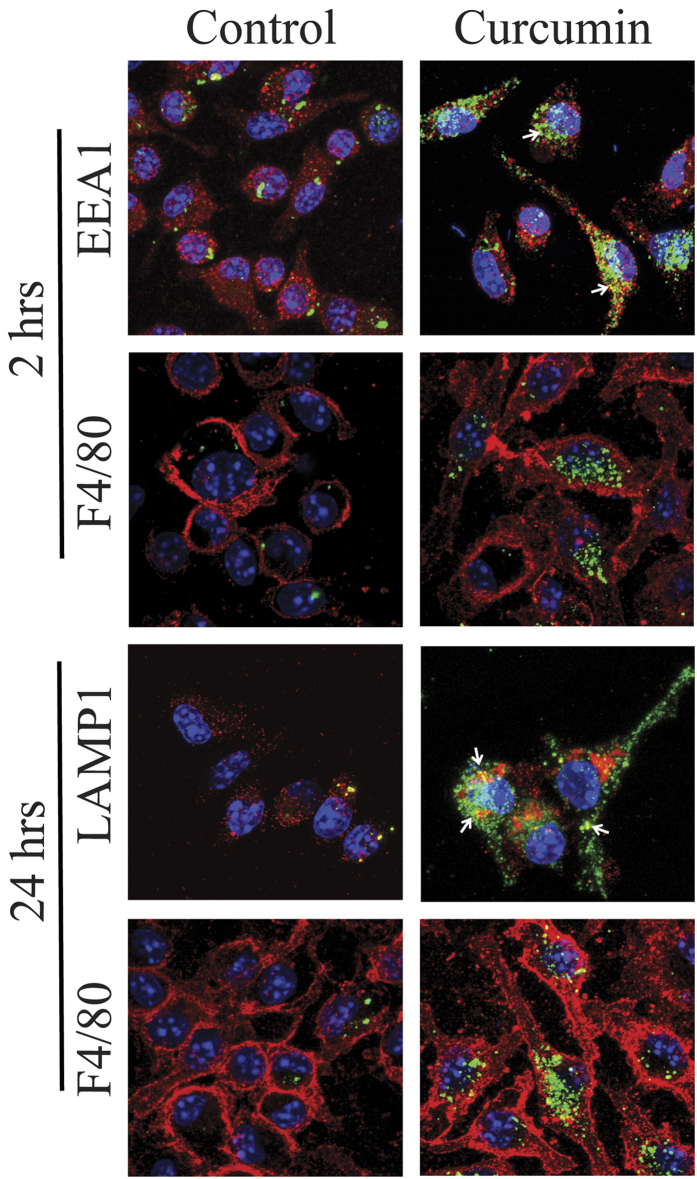
Curcumin treated macrophages from FAP mice (hTTR V30M/Hsf mice) present increased internalization and degradation of TTR aggregates. Representative pictures of double immunofluorescence labeling for TTR (green) and EEA1 (red), TTR (green) and F4/80 (red) or TTR (green) and Lamp-1 (red), denoting TTR intracellular aggregates co- localizing with endosomes and lysosomes in curcumin treated mice (arrows). Superposition of the labels, with DAPI (blue), is shown. 63× magnification. Data presented is representative of three independent experiments. Treated macrophages were pre-exposed to curcumin before addition of TTR aggregates to cell culture medium and, by double immunofluorescence confocal microscopy, we found that pre- exposition of macrophages with curcumin resulted in rapid internalization of TTR extracellular aggregates and lysosomal degradation as shown by co-localization of TTR oligomers and EEA1 (t = 2 h, Fig. 7) or LAMP-1 (t = 24 h, Fig. 7), respectively.

Curcuminoids are known to improve the function of the innate immune system by increasing clearance of aggregates in several amyloid diseases^23^,^24^. Therefore, we further investigated the effect of curcumin in bone marrow derived macrophages exposed to TTR aggregates. Macrophage culture purity was confirmed for all time points analyzed, by staining with F4/80, a well-characterized and extensively referenced mature macrophage marker^25^,^26^. In control macrophages (treated with vehicle) uptake of extracellular TTR aggregates was scarce (Fig. 7). In addition, intracellular transport of TTR aggregates progressed very slowly as shown by limited co-localization between those and EEA1 (t = 2 h, Fig. 7) or LAMP-1 (t = 24 h, Fig. 7).

Treated macrophages were exposed to curcumin before addition of TTR aggregates to the cell culture medium. Using confocal microscopy, we found that curcumin stimulation resulted in rapid internalization of TTR aggregates and lysosomal degradation as shown by co-localization with EEA1 (t = 2 h, Fig. 7) and LAMP-1 (t = 24 h, Fig. 7), respectively.

The optimal dose of curcumin (1 μM) was determined using a wide range of concentrations (0.01–100 μM). Concentrations higher than 1 μM did not increase aggregates internalization by macrophages.

## Discussion

The working hypothesis for the TTR amyloid cascade, supported by extensive work and literature, led to the notion that stabilizing TTR native structure could be a promising therapeutic approach to stop the production of toxic TTR amyloidogenic species. Since FAP and other TTR-related amyloidoses are progressive and complex multi-system disorders, therapeutic strategies aiming multiple molecular targets simultaneously, either through combination therapies or multi-target-directed compounds, could improve therapy efficacy and treatment outcome.

Curcumin has a symmetric and extended conjugated molecular structure that resembles the structure of Congo red, the classic gold standard for detecting amyloid deposits^21^. In addition, curcumin is able to efficiently cross the blood-brain barrier, which is an essential feature for therapies aiming amyloid deposition in central nervous system^27^,^28^. Concerning TTR amyloidosis, previous work from our group has shown that, *in vitro*, curcumin binding to the largely unoccupied thyroxin binding pockets in the native TTR structure increases its resistance to dissociation in non-native monomers that polymerize into toxic aggregates^12^. Very recently, the crystal structure of the complex formed between TTR and curcumin has been determined and it corroborates our initial findings by showing that intact curcumin binds at the two T_4_ binding sites located in the TTR tetramer central channel^29^.

In the present study, we investigated the effect of curcumin supplementation in a late-stage mouse model for FAP. After gut absorption and systemic distribution, curcumin selectively interacted with TTR in plasma, in the largely unoccupied T_4_ binding pockets, increasing TTR stability. In addition, curcumin administration significantly lowered TTR non-fibrillar deposition and toxicity in tissues, in particular along the gastrointestinal tract and DRG. These results confirm the previous study in early stage FAP mice^13^ and are in accordance with the working hypothesis for TTR amyloidosis by which stabilizing TTR native fold by small molecules would block protein aggregation and toxicity^30^.

Moreover, after Congo red birefringence analysis of stomach sections, we found that curcumin treated mice presented fewer and noticeably smaller congophilic deposits as compared to control mice. These results were supported by a substantial reduction on the levels of MMP-9 in treated mice and clearly indicate that curcumin promotes amyloid remodeling, reabsorption and extracellular matrix recovery. Our observations corroborate the results obtained for other amyloidoses since, for instance, administration of dietary curcumin to aged mouse models of AD resulted in labeling Aβ plaques and reducing amyloid levels and plaque burden^28^,^31^.

Though our data indicates that curcumin modulates the TTR cascade by direct interaction with TTR aggregate intermediates, we speculate whether the diverse array of molecular targets for curcumin might potentiate its neuroprotective effect and improve treatment outcome *in vivo*. For instance, since high concentrations of Zn^2+^ and Cu^2+^ can trigger TTR amyloid formation^32^, it seems reasonable to speculate whether curcumin known ability to form complexes with these ions^17^ might contribute to inhibit abnormal TTR aggregation and toxicity *in vivo*. Moreover, compelling evidence indicates that curcumin is able to suppress inflammation through multiple signaling pathways^33^,^34^. Thus, reduction of the pro-inflammatory response to TTR deposition in DRG, observed in curcumin treated mice, might be attributed not only to curcumin anti-amyloidogenic effects but also to its well-known capacity to block the production of key mediators of inflammation such as NF-kB, TNF-α, or IL-1β^33^. However, curcumin has been recognized mainly as an anti-oxidant compound^35^,^36^,^37^. In accordance, a recent study by Moustapha *et al*.^38^ revealed that this is the predominant effect at very low doses of curcumin (lower than 1 μM) while at higher concentrations, 10 μM, it starts inducing early events of autophagy and at even higher dosages (>25 μM) it induces apoptosis and endoplasmic reticulum stress causing calcium release, with destabilization of the mitochondrial compartment and promoting tumor cell death^38^,^39^. This hormetic characteristic of curcumin has been detailed using a tumor cell line namely, hepatocellular carcinoma Huh-7 cells^38^. Nonetheless, molecular effects of curcumin on complex cell systems might most likely be cell-type specific in a concentration and time-dependent manner.

Defects in the autophagic machinery or mitochondrial dynamics might play a pathological role in neurodegenerative disorders^40^ and might also be impaired in FAP. In fact, it was recently described that curcumin is able to decrease monomeric TTR, by promoting LC3 cleavage, thus enhancing autophagy, in a cell culture system^41^. Importantly, studies of Hsf-1 knock-out mice suggest that *Hsf-1* might play a role in the regulation of autophagy activity^42^. Therefore, future studies disclosing the role of autophagy in FAP should also encompass the use of other animal models.

Transthyretin, either wild-type or mutant, can be internalized by diverse types of cells, including hepatocytes, mouse embryonic fibroblasts, yolk sac cells, sensory neurons and glial cells^43^,^44^,^45^,^46^. Recent work from Misumi and colleagues has shown that fibroblasts endocyte and degrade TTR aggregates *in vitro* and *in vivo*^47^. All together these studies provide evidence for the complex cellular dynamics underlying TTR catabolism and clearance and its impact in the FAP pathology. We speculate that cellular impairment of one or more of these mechanisms might shift the amyloid formation/degradation equilibrium towards pathogenic TTR aggregate accumulation in the extracellular matrix. Moreover, TTR aggregates internalization might also be deleterious to cells leading to death and organ dysfunction, a topic that will need further investigation.

During the last years, considerable focus has been given to the role of the immune system on amyloid pathologies. Macrophages and microglia are the innate immune cells responsible for clearance of pathogens and waste products. Curcuminoids have been reported to enhance phagocytosis of Aβ by upregulating the transcription of 1,4-mannosyl-glycoprotein 4-β-N- acetyl glucosaminyl transferase and other genes, including Toll-like receptors, typically down-regulated in mononuclear cells of AD patients^19^. Similarly, we found that pre-exposition of macrophages isolated from aged hTTR V30M/Hsf mice, to physiologically achievable doses of curcumin (1–10 μM), improved their phagocytic uptake and degradation of extracellular TTR amyloid aggregates compared to control macrophages (exposed to vehicle). Thus, our observations indicate that aged FAP macrophages present deficient phagocytic ability, suggesting a possible impairment in their functions. In fact, it has been previously demonstrated that nerve biopsies from FAP patients did not display innate cellular infiltrate surrounding amyloid deposits, as should be expected^5^ and that macrophage density is downregulated in a FAP mouse model after an injured stimulus^26^.

Despite the molecular mechanisms underlying the clearance impairment of macrophage are mostly unknown, the current study shows that chronic administration of curcumin may modulate the innate immune system response to extracellular accumulation of TTR and provide a previously uncharacterized approach for FAP therapy. Future studies will further address the role of macrophages in FAP pathology using different FAP mouse lines at different stages of TTR amyloid formation.

Currently, a diverse array of emerging disease-modifying agents is under analysis in human clinical trials^38^. From TTR stabilizers (diflunisal, tafamidis), gene therapies to suppress TTR expression (siRNAs) and amyloid fibrils disruptors (doxycycline/TUDCA), the perspectives for an effective therapy for TTR amyloidosis seem more encouraging nowadays than ever before. Nevertheless, much is still unknown regarding long-term safety and efficacy of such approaches and, with the exception of doxycycline/TUDCA therapy, most of them target only early stages of the disease.

Our results suggest curcumin as an interesting compound for late-stage TTR amyloidosis. Taking into account its pluripotency, high tolerability, long history of use, and inexpensive cost, we speculate that its combined administration with available therapies might boost treatment efficacy and outcome for advanced amyloidosis.

In conclusion, the present work demonstrates that curcumin inhibits TTR aggregation in a dose dependent manner and enhance TTR aggregates clearance by macrophages *in vitro*. When administrated to aged FAP mice, curcumin not only reduced TTR burden and toxicity but also remodeled congophilic material in tissues. Overall, our results clearly support curcumin or optimized derivatives as promising multi-target disease-modifying agent for late-stage amyloidosis.

## Materials and Methods

### Curcumin

Curcumin was purchased from Sigma-Aldrich, St. Louis, MO, USA. Curcumin had ≥80% purity (HPLC), with total curcuminoids ≥94% (HPLC).

### Production of recombinant TTR variants

Recombinant TTR V30M and TTR Y78F production, isolation and purification were performed as previously described^48^.

### Preparation of fluorescent TTR oligomers

Soluble TTR V30M in PBS was filtered through 0.2 μm Anotop syringe filters (Whatman, England) and labeled with the fluorescent dye Alexa Fluor 488 (Invitrogen, Carlsbad, CA, USA) according to the manufacturer’s instructions. TTR aggregates were generated by incubating the protein (2 mg/ml), with stirring, at room temperature for 7 days^6^. Preparations were analyzed by dynamic light scattering (DLS) at 25 °C in a Malvern Zetasizer Nano ZS (Malvern, Worcestershire, UK) as previously reported, to confirm TTR pathogenic aggregation^11^.

### Cell culture assays to assess ER stress and apoptosis

Soluble TTR was dissolved at 2 mg/ml in PBS and curcumin stocks were prepared in dimethyl sulfoxide (DMSO). Soluble TTR was mixed with different concentrations of curcumin (0.02–200 μM) or solvent alone (DMSO). A sample containing only curcumin 200 μM was also prepared. All samples were kept in Eppendorf amber tubes (for light protection) and incubated for 6 days at 37 °C to allow aggregation. These samples were then diluted (18×) in cell culture medium to attain a concentration of 2 μM TTR and increasing concentrations of curcumin ranging 0.001–11 μM. Following, these samples were added to the cells as following described.

Rat Schwannoma cells (RN22) (American type Cell Collection) were propagated and maintained as described previously^11^. Briefly, 80% confluent cells in Dulbecco’s minimal essential medium (DMEM) supplemented with 1% fetal bovine serum were exposed, for 24 h, with: i) vehicle (DMSO); ii) 2 μM of freshly prepared soluble TTR; and the diluted samples prepared above, namely iii) TTR Y78F oligomers or iv) curcumin-induced TTR oligomers or v) 11 μM of curcumin alone. After treatment, cells were trypsinized and lysed using ice-cold lysis buffer containing 5 mM ethylenediamine tetraacetic acid, 2 mM ethylene glycol tetra acetic acid, 20 mM 3-(N-morpholino) propane sulfonic acid, 1% Triton X-100, 1 mM phenyl methane sulfonyl fluoride (PMSF) and a protease inhibitor mix (GE Healthcare). Cell lysates were used for determination of BiP intracellular levels and caspase-3 activity. Protein concentration in lysates was determined using a Bio-Rad protein assay kit. Caspase-3 activity was accessed using the CaspACE fluorimetric 96-well plate assay system (Sigma- Aldrich, St. Louis, MO, USA) according to the manufacturer’s instructions. For determination of BiP levels by western blot analysis equal amounts of protein from lysates were separated in 15% SDS-PAGE and transferred onto a nitrocellulose Hybond-C membrane (Amersham Biosciences) using a semi-dry system. The primary antibodies and the respective dilutions used were: rabbit polyclonal anti-BiP (1:1000) and mouse monoclonal anti-GAPDH (1:3000) (Abcam, Cambridge, UK). Detection was performed with Luminata^TM^ Crescendo (enhanced chemiluminescence, Millipore, Billerica, MA). Quantification of blots was performed with a Bio-Rad ChemiDoc XRS system using the IMAGELAB software and immunosignals were normalized with GAPDH expression. Results are presented as normalized density ± Standard Error of the Mean (SEM).

### Ethics statement

All the experiments described herein were approved by the Portuguese General Veterinarian Board (authorization number 024976 from DGV-Portugal) and are in compliance with national rules and the European Communities Council Directive (2010/63/EU), for the care and handling of laboratory animals.

### FAP transgenic mouse model

Aged transgenic mice for human TTR V30M in a TTR null background, heterozygous for the heat shock transcription factor 1 (*Hsf-1*), labeled hTTR V30M/Hsf mice, were used for the experiments^8^. Animals were housed in pathogen-free conditions, in a controlled-temperature room, maintained under a 12 h light/dark period, with water and food *ad libitum*. Treated animals were given standard mouse chow containing 2% (w/w) curcumin (Sigma-Aldrich, St. Louis, MO, USA) starting at age 14 months for 6 weeks (treated, *n* = 12). Control animals were fed regularly (*n* = 14). Mice were euthanized at 15.5 months of age with a solution containing ketamine and medetomidine. Blood samples were collected from the vena cava, plasma was separated by centrifugation and stored at −20 °C prior to analysis. Mice tissues, in particular the gastrointestinal tract (stomach, duodenum and colon) and the dorsal root ganglia (DRG) were immediately excised and frozen at −80 °C or fixed in 10% neutral buffered formalin for western blot or light microscopy analysis, respectively.

### Determination of TTR levels in mice plasma

Transthyretin levels in mice plasma were quantified by radial immunodiffusion (RID) using the Human Prealbumin Bindarid, Radial Immunodiffusion Kit (The Binding Site, Birmingham, UK) following the manufacturer’s instructions.

### Thyroxine (T_4_) binding gel electrophoresis

Five microliters of plasma from control and curcumin treated mice were incubated with [^125^I]T_4_ (specific radioactivity 1250 μCi/μg; Perkin–Elmer, MA, USA). Plasma proteins were separated by native PAGE^49^. The gel was dried, subjected to phosphor imaging (Typhoon 8600; Molecular Diagnostics, Amersham Biosciences) and analyzed using the ImageQuant program version 5.1.

### Isoelectric focusing (IEF) in semi-denaturing conditions

Isoelectric focusing (IEF) of plasma TTR was performed as detailed elsewhere^49^. Thirty microliters of plasma from control and curcumin treated mice were subjected to native electrophoresis (PAGE). The TTR gel band was excised and applied to a semi-denaturing (4 M urea) pH 4–6.5 gradient isoelectric focusing (IEF) gel run for 6 h at 1200 V. Proteins were stained with Coomassie Blue. The gels were scanned and subjected to densitometry analysis using the ImageQuant program version 5.1.

### Semi-quantitative immunohistochemistry (SQ-IHC) and Congo red staining

Immunohistochemistry and histological analyses of mice GI tract and DRG were performed as described previously^50^. Tissue sections (5 μm thick) were deparaffinated in histoclear and rehydrated in a descent alcohol series. Endogenous peroxidase activity was quenched with 3% hydrogen peroxide in methanol and sections were blocked in 10% fetal bovine serum, 1% bovine serum albumin and 0.5% Triton X-100 in PBS. The primary antibodies were: rabbit polyclonal anti-TTR (1:1000) (DAKO, Denmark), goat polyclonal anti-BiP (1:50), rabbit polyclonal anti-Fas death receptor (1:200), goat polyclonal anti-IL-1β (1:25), goat polyclonal anti-TNF-α (1:25), rabbit polyclonal anti-NF-κB p65 (1:25) (Santa Cruz Biotechnology, Santa Cruz, CA, USA), rabbit polyclonal anti-cleaved caspase-3 (1:100, R&D Systems, Minneapolis, MN) which were diluted in blocking solution and incubated overnight at 4 °C. Antigen visualization was performed with the biotin–extravidin peroxidase ABC kit (Vector, Burlingame, CA) using hydrogen peroxide and diaminobenzidine as substrate and chromogen, respectively. Semi-quantitative immunohistochemical (SQ-IHC) analysis was performed using Image-Pro Plus version 5.1 software. This application enables the measurement of the area occupied by pixels corresponding to the immunohistochemistry substrate’s color that is normalized relatively to the total area. The area occupied by chromogen represents the total amount of antigen present and all brown staining was automated quantified by the image analysis software. Each slide was analyzed in five different representative areas at 20x magnification.

Tissues were also stained with Congo red and observed under polarized light for the detection of amyloid deposits^21^. Briefly, deparaffinated tissue sections were incubated for 20 min with 0.01% NaOH in 80% ethanol saturated with NaCl followed by staining with 0.5% Congo red in the previous solution. Then, preparations were washed with tap water, stained with hematoxilin and analyzed under polarized light. Amyloid was identified by distinctive apple green birefringence. All image analyses were carried out independently by two investigators unaware of the origin of the tested tissue sections.

### Total protein extracts and Western blot analysis

Stomach and DRG were homogenized on ice in a small glass rod homogenizer with lysis buffer containing 5 mM EDTA, 2 mM EGTA, 20 mM MOPS, 1% Triton X-100, 1 mM PMSF and Protease Inhibitor Mix (GE Healthcare). After centrifugation (14,000 rpm for 20 min at 4 °C) protein concentration in the supernatant was determined by the Bradford protein assay (Bio-Rad, CA, USA). Western blotting was performed as previously described^17^. Protein lysates were run on 15% SDS-PAGE (50 μg/lane) and transferred to a nitrocellulose Hybond-C membrane using a Mini Trans-Blot Cell (Bio-Rad) system. Membranes were incubated overnight at 4 °C with the primary antibodies: rabbit polyclonal anti-TTR (1:1000) (DAKO, Denmark), rabbit polyclonal anti-BiP (1:1000) and mouse monoclonal anti- glyceraldehyde 3-phosphate dehydrogenase (GAPDH) (1:3000) (Abcam, Cambridge, UK). Detection was performed with Luminata^TM^ Crescendo (enhanced chemiluminescence, Millipore, Billerica, MA). Quantification of blots was performed with a Bio-Rad ChemiDoc XRS system using the IMAGELAB software and immunosignals were normalized to GAPDH expression. Results are presented as normalized density ± SEM.

### Culture of primary bone-marrow derived macrophages

Bone marrow-derived macrophages were obtained from femurs and tibias of 14 months-old hTTR V30M/Hsf mice and were cultured at 4 × 10^6^ cells per petri dish for 6 days (bacterial plates; Sterilin) in 8 mL of RPMI 1640 (Lonza) supplemented with 10% heat-inactivated fetal bovine serum, 1% sodium pyruvate, 1% HEPES, 1% L-glutamine (GIBCO), 0.05 mM 2- mercaptoethanol (Sigma-Aldrich) and 20% L929 cell-conditioned media (LCCM), as previously described^51^. At day 6, macrophages were harvested and seeded into 24-well tissue culture plates (Orange Scientific, Braine-l′Alleud, Belgium) at 0.5 × 10^6^ cells/mL. Cells were rested and stimulated at day 7, overnight in the absence (control) or presence of curcumin (final concentration in the medium 0.01 μM to 10 μM). Oligomeric TTR prepared as described above was then added to the medium at appropriate dilution (2 μg/mL). After different incubation periods (2 and 24 h), cells were washed with PBS and fixed with 4% ice-cold paraformaldehyde for subsequent confocal microscopy analysis.

### Confocal Laser Microscopy

For mouse cell imaging by fluorescence analysis, cells were plated over a glass coverslip lipopolysaccharide (LPS) free, in a 24 well plate, and fixed with 4% paraformaldehyde. After permeabilization, fixed cells were blocked with phosphate buffer supplemented with 10% fetal bovine serum and 0.5% Triton X-100 for 1 h at room temperature and incubated overnight at 4 °C with rabbit polyclonal antibody against Early Endosome Antigen 1 (EEA1, 1:250, Sigma-Aldrich) to localize early endosomes, mouse anti- Lysosomal-Associated Membrane Protein 1 (LAMP- 1, 1:75, Abcam) as a marker for lysosomes and rat monoclonal anti-F4/80 (1:100, Serotec), as a macrophage marker. For detection of primary antibodies, anti-rabbit Alexa Fluor 568, anti-rat Alexa Fluor 594 or anti-mouse Alexa Fluor 568 (1:1,000; Molecular Probes, Invitrogen) were incubated for 1 h at room temperature. Coverslips were mounted in Vectashield with DAPI (Vector Laboratories) and visualized under a Laser Scanning Confocal microscope Leica SP5.

## Additional Information

**How to cite this article**: Ferreira, N. *et al*. Curcumin: A multi-target disease-modifying agent for late-stage transthyretin amyloidosis. *Sci. Rep.* **6**, 26623; doi: 10.1038/srep26623 (2016).

## Supplementary Material

Supplementary Information

## References

[b1] Kirschner D. A., Abraham C. & Selkoe D. J. X-ray diffraction from intraneuronal paired helical filaments and extraneuronal amyloid fibers in Alzheimer disease indicates cross-beta conformation. Proc. Natl. Acad. Sci. USA 83, 503–507 (1986). 3455785 10.1073/pnas.83.2.503 PMC322888

[b2] Lorenzo A. & Yankner B. A. Beta-amyloid neurotoxicity requires fibril formation and is inhibited by congo red. Proc. Natl. Acad. Sci. USA 91, 12243–12247 (1994). 7991613 10.1073/pnas.91.25.12243 PMC45413

[b3] Deshpande A., Mina E., Glabe C. & Busciglio J. Different conformations of amyloid beta induce neurotoxicity by distinct mechanisms in human cortical neurons. J. Neurosci. 26, 6011–6018 (2006). 16738244 10.1523/JNEUROSCI.1189-06.2006 PMC6675207

[b4] Saraiva M. J., Magalhaes J., Ferreira N. & Almeida M. R. Transthyretin deposition in familial amyloidotic polyneuropathy. Curr. Med. Chem. 19, 2304–2311 (2012). 22471982 10.2174/092986712800269236

[b5] Sousa M. M. . Familial amyloid polyneuropathy: receptor for advanced glycation end products-dependent triggering of neuronal inflammatory and apoptotic pathways. J. Neurosci. 21, 7576–7586 (2001). 11567048 10.1523/JNEUROSCI.21-19-07576.2001 PMC6762914

[b6] Teixeira P. F., Cerca F., Santos S. D. & Saraiva M. J. Endoplasmic reticulum stress associated with extracellular aggregates. Evidence from transthyretin deposition in familial amyloid polyneuropathy. J. Biol. Chem. 281, 21998–22003 (2006). 16751191 10.1074/jbc.M602302200

[b7] Sousa M. M., do Amaral J. B., Guimarães A. & Saraiva M. J. Up-regulation of the extracellular matrix remodeling genes, biglycan, neutrophil gelatinase-associated lipocalin, and matrix metalloproteinase-9 in familial amyloid polyneuropathy. FASEB J. 19, 124–126 (2005). 15536164 10.1096/fj.04-2022fje

[b8] Santos S. D., Fernandes R. & Saraiva M. J. The heat shock response modulates transthyretin deposition in the peripheral and autonomic nervous systems. Neurobiol. Aging. 31, 280–289 (2010). 18485534 10.1016/j.neurobiolaging.2008.04.001

[b9] Koike H. . Pathology of early- vs late-onset TTR Met30 familial amyloid polyneuropathy. Neurology. 63, 129–138 (2004). 15249622 10.1212/01.wnl.0000132966.36437.12

[b10] Dubrey S., Ackermann E. & Gillmore J. The transthyretin amyloidoses: advances in therapy. Postgrad. Med. J. 91, 439–448 (2015). 26048914 10.1136/postgradmedj-2014-133224

[b11] Ferreira N. . Binding of epigallocatechin-3-gallate to transthyretin modulates its amyloidogenicity. FEBS Lett. 583, 3569–3576 (2009). 19861125 10.1016/j.febslet.2009.10.062

[b12] Ferreira N., Saraiva M. J. & Almeida M. R. Natural polyphenols inhibit different steps of the process of transthyretin (TTR) amyloid fibril formation. FEBS Lett. 585, 2424–2430 (2011). 21740906 10.1016/j.febslet.2011.06.030

[b13] Ferreira N., Santos S. A., Domingues M. R., Saraiva M. J. & Almeida M. R. Dietary curcumin counteracts extracellular transthyretin deposition: insights on the mechanism of amyloid inhibition. Biochim. Biophys. Acta. 1832, 39–45 (2013). 23069388 10.1016/j.bbadis.2012.10.007

[b14] Singh P. K. . Curcumin modulates α-synuclein aggregation and toxicity. ACS Chem. Neurosci. 4, 393–407 (2013). 23509976 10.1021/cn3001203 PMC3605819

[b15] Thapa A., Jett S. D. & Chi E. Y. Curcumin Attenuates Amyloid-β Aggregate Toxicity and Modulates Amyloid-β Aggregation Pathway. ACS Chem. Neurosci. 7, 56–68 (2016). 26529184 10.1021/acschemneuro.5b00214

[b16] Menon V. P. & Sudheer A. R. Antioxidant and anti-inflammatory properties of curcumin. Adv. Exp. Med. Biol. 595, 105–125 (2007). 17569207 10.1007/978-0-387-46401-5_3

[b17] Baum L. & Ng A. Curcumin interaction with copper and iron suggests one possible mechanism of action in Alzheimer’s disease animal models. J. Alzheimers Dis. 6, 367–377 (2004). 15345806 10.3233/jad-2004-6403

[b18] Chandra V. . Incidence of Alzheimer’s disease in a rural community in India: the Indo-US study. Neurology 57, 985–989 (2001). 11571321 10.1212/wnl.57.6.985

[b19] Fiala M. . Innate immunity and transcription of MGAT-III and Toll-like receptors in Alzheimer’s disease patients are improved by bisdemethoxycurcumin. Proc. Natl. Acad. Sci. USA 104, 12849–12854 (2007). 17652175 10.1073/pnas.0701267104 PMC1937555

[b20] Lee A. S. The ER chaperone and signaling regulator GRP78/BiP as a monitor of endoplasmic reticulum stress. Methods 35, 373–381 (2005). 15804610 10.1016/j.ymeth.2004.10.010

[b21] Puchtler H. & Sweat F. Congo red as a stain for fluorescence microscopy of amyloid. J. Histochem. Cytochem. 13, 693–694 (1965). 4160077 10.1177/13.8.693

[b22] Macedo B., Batista A. R., Ferreira N., Almeida M. R. & Saraiva M. J. Anti-apoptotic treatment reduces transthyretin deposition in a transgenic mouse model of Familial Amyloidotic Polyneuropathy. Biochim. Biophys. Acta. 1782, 517–522 (2008). 18572024 10.1016/j.bbadis.2008.05.005

[b23] Zhang L. . Curcuminoids enhance amyloid-beta uptake by macrophages of Alzheimer’s disease patients. J. Alzheimers Dis. 10, 1–7 (2006). 16988474 10.3233/jad-2006-10101

[b24] Fiala M. Re-balancing of inflammation and abeta immunity as a therapeutic for Alzheimer’s disease-view from the bedside. CNS Neurol. Disord. Drug. Targets 9, 192–196 (2010). 20205641 10.2174/187152710791012044

[b25] Khazen W. . Expression of macrophage-selective markers in human and rodent adipocytes. FEBS Lett. 579, 5631–5634 (2005). 16213494 10.1016/j.febslet.2005.09.032

[b26] Gonçalves N. P., Teixeira-Coelho M. & Saraiva M. J. The inflammatory response to sciatic nerve injury in a familial amyloidotic polyneuropathy mouse model. Exp. Neurol. 257, 76–87 (2014). 24800914 10.1016/j.expneurol.2014.04.030

[b27] Ryu E. K., Choe Y. S., Lee K. H., Choi Y. & Kim B. T. Curcumin and dehydrozingerone derivatives: synthesis, radiolabeling, and evaluation for beta-amyloid plaque imaging. J. Med. Chem. 49, 6111–6119 (2006). 17004725 10.1021/jm0607193

[b28] Yang F. . Curcumin inhibits formation of amyloid beta oligomers and fibrils, binds plaques, and reduces amyloid *in vivo*. J. Biol. Chem. 280, 5892–5901 (2005). 15590663 10.1074/jbc.M404751200

[b29] Ciccone L., Tepshi L., Nencetti S. & Stura E. A. Transthyretin complexes with curcumin and bromo-estradiol: evaluation of solubilizing multicomponent mixtures. N Biotechnol. 32, 54–64 (2015). 25224922 10.1016/j.nbt.2014.09.002

[b30] Ueda M. & Ando Y. Recent advances in transthyretin amyloidosis therapy. Transl Neurodegener 3, 19 (2014). 25228988 10.1186/2047-9158-3-19 PMC4165622

[b31] Garcia-Alloza M., Borrelli L. A., Rozkalne A., Hyman B. T. & Bacskai B. J. Curcumin labels amyloid pathology *in vivo*, disrupts existing plaques, and partially restores distorted neurites in an Alzheimer mouse model. J. Neurochem. 102, 1095–1104 (2007). 17472706 10.1111/j.1471-4159.2007.04613.x

[b32] Wilkinson-White L. E. & Easterbrook-Smith S. B. Characterization of the binding of Cu(II) and Zn(II) to transthyretin: effects on amyloid formation. Biochemistry 46, 9123–9132 (2007). 17630783 10.1021/bi700607z

[b33] Aggarwal B. B., Gupta S. C. & Sung B. Curcumin: an orally bioavailable blocker of TNF and other pro-inflammatory biomarkers. Br. J. Pharmacol. 169, 1672–1692 (2013). 23425071 10.1111/bph.12131 PMC3753829

[b34] Morales I., Guzmán-Martínez L., Cerda-Troncoso C., Farías G. A. & Maccioni R. B. Neuroinflammation in the pathogenesis of Alzheimer’s disease. A rational framework for the search of novel therapeutic approaches. Front. Cell. Neurosci. 8, 112 (2014). 24795567 10.3389/fncel.2014.00112 PMC4001039

[b35] Balasubramanyam M. . Curcumin-induced inhibition of cellular reactive oxygen species generation: novel therapeutic implications. J. Biosci. 28, 715–721 (2003). 14660871 10.1007/BF02708432

[b36] Chan W. H., Wu H. J. & Hsuuw Y. D. Curcumin inhibits ROS formation and apoptosis in methylglyoxal-treated human hepatoma G2 cells. Ann. N. Y. Acad. Sci. 1042, 372–378 (2005). 15965083 10.1196/annals.1338.057

[b37] Jain A. . Curcumin inhibits PhIP induced cytotoxicity in breast epithelial cells through multiple molecular targets. Cancer Lett. 365, 122–131 (2015). 26004342 10.1016/j.canlet.2015.05.017 PMC7742852

[b38] Moustapha A. . Curcumin induces crosstalk between autophagy and apoptosis mediated by calcium release from the endoplasmic reticulum, lysosomal destabilization and mitochondrial events. Cell Death Discovery 1, 15017, doi: 10.1038/cddiscovery.2015.17 (2015). PMC4979459 27551451

[b39] Rainey N., Motte L., Aggarwal B. B. & Petit. P. X. Curcumin hormesis mediates a cross-talk between autophagy and cell death. Cell Death Dis 3, 6:e2003 (2015). 10.1038/cddis.2015.343 PMC4720879 26633709

[b40] Galluzzi L., Kepp O., Trojel-Hansen C. & Kroemer G. Mitochondrial control of cellular life, stress, and death. Circ. Res. 111, 1198–1207 (2012). 23065343 10.1161/CIRCRESAHA.112.268946

[b41] Li H. . Curcumin could reduce the monomer of TTR with Tyr114Cys mutation via autophagy in cell model of familial amyloid polyneuropathy. Drug Des. Devel. Ther. 8, 2121–2128 (2012). 10.2147/DDDT.S70866 PMC4222630 25382970

[b42] Tong Z. . HSF-1 is involved in attenuating the release of inflammatory cytokines induced by LPS through regulating autophagy. Shock. 41, 449–453 (2014). 24430550 10.1097/SHK.0000000000000118

[b43] Sousa M. M. . Evidence for the role of megalin in renal uptake of transthyretin. J. Biol. Chem. 275, 38176–38181 (2000). 10982792 10.1074/jbc.M002886200

[b44] Sousa M. M. & Saraiva M. J. Internalization of transthyretin. Evidence of a novel yet unidentified receptor-associated protein (RAP)-sensitive receptor. J. Biol. Chem. 276, 14420–14425 (2001). 11278770 10.1074/jbc.M010869200

[b45] Fleming C. E., Mar F. M., Franquinho F., Saraiva M. J. & Sousa M. M. Transthyretin internalization by sensory neurons is megalin mediated and necessary for its neuritogenic activity. J. Neurosci. 29, 3220–3232 (2009). 19279259 10.1523/JNEUROSCI.6012-08.2009 PMC6666452

[b46] Gonçalves N. P., Costelha S. & Saraiva M. J. Glial cells in familial amyloidotic polyneuropathy. Acta Neuropathol. Commun. 2, 177 (2014). 25519307 10.1186/s40478-014-0177-8 PMC4280682

[b47] Misumi Y., Ando Y., Gonçalves N. P. & Saraiva M. J. Fibroblasts endocytose and degrade transthyretin aggregates in transthyretin-related amyloidosis. Lab. Invest. 93, 911–920 (2013). 23817086 10.1038/labinvest.2013.83

[b48] Furuya H. . Production of recombinant human transthyretin with biological activities toward the understanding of the molecular basis of familial amyloidotic polyneuropathy (FAP). Biochemistry. 30, 2415–2421 (1991). 1848097 10.1021/bi00223a017

[b49] Almeida M. R. . Selective binding to transthyretin and tetramer stabilization in serum from patients with familial amyloidotic polyneuropathy by an iodinated diflunisal derivative. Biochem. J. 381, 351–356 (2004). 15080795 10.1042/BJ20040011 PMC1133839

[b50] Ferreira N., Saraiva M. J. & Almeida M. R. Epigallocatechin-3-gallate as a potential therapeutic drug for TTR-related amyloidosis: “*in vivo*” evidence from FAP mice models. PLos One. 7, e29933 (2012). 22253829 10.1371/journal.pone.0029933 PMC3254632

[b51] McNab F. W. . Type I IFN induces IL-10 production in an IL-27-independent manner and blocks responsiveness to IFN-γ for production of IL-12 and bacterial killing in Mycobacterium tuberculosis-infected macrophages. J. Immunol. 193, 3600–3612 (2014). 25187652 10.4049/jimmunol.1401088 PMC4170673

