# Supplementary material for: Curcumin: A multi-target disease-modifying agent for late-stage transthyretin amyloidosis
Source: Sci Rep. 2016 May 20;6:26623. doi: 10.1038/srep26623 (PMC4873750; doi:10.1038/srep26623)

SUPPLEMENTARY DATA

Curcumin: A multi-target disease-modifying agent for late-stage transthyretin amyloidosis

Nelson Ferreira[1,2], Nádia P. Gonçalves[1,2,3], Maria J. Saraiva[1,2,3], Maria R. Almeida[1,2,3]

[1] IBMC - Instituto de Biologia Molecular e Celular, Universidade do Porto, Rua Alfredo Allen, 208, 4200 – 135 Porto, Portugal

[2] i3S – Instituto de Investigação e Inovação em Saúde da Universidade do Porto, Rua Alfredo Allen, 208, 4200 – 135 Porto, Portugal

[3] ICBAS, Instituto de Ciências Biomédicas Abel Salazar, Universidade do Porto, Rua Jorge Viterbo Ferreira 228, 4050 – 313 Porto, Portugal

To whom correspondence should be addressed:

Maria Rosário Almeida

IBMC - Instituto de Biologia Molecular e Celular
i3S – Instituto de Investigação e Inovação em Saúde da Universidade do Porto,
Rua Alfredo Allen, 208, 4200 – 135 Porto, Portugal
Phone: +35122040800
Email: ralmeida@ibmc.up.pt

SUPPLEMENTARY DATA

Supp. Fig. 1 - Curcumin binds to TTR in plasma. A. Representative PAGE analysis of [^{125}I]-T_4 distribution among T_4 binding proteins after incubation with plasma from curcumin treated and control hTTR V30M/Hsf mice. Plasma T_4 binding proteins are indicated. **B.** The histogram shows percentage of total bound [^{125}I]-T_4 to each plasma T_4 binding protein. (***p < 0.001).

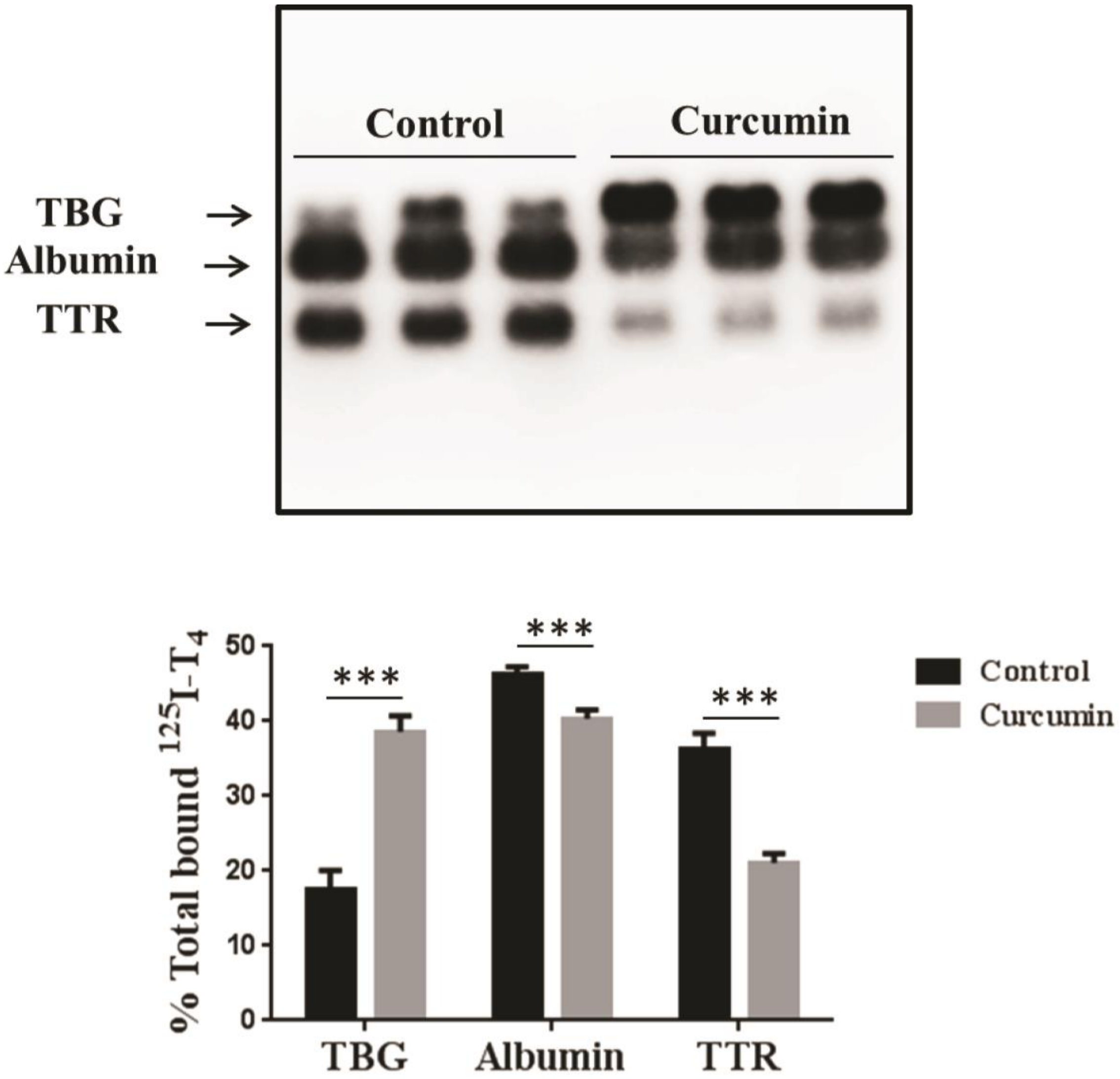

Supp. Fig. 2 - Curcumin increases TTR resistance to dissociation. A. Plasmas from mice treated with curcumin and controls were subjected to isoelectric focusing analysis (IEF) under semi-dissociating conditions. Different TTR molecular species are indicated. **B.** The histogram shows TTR tetramer/total TTR ratio obtained after densitometry analysis of IEF gels for both curcumin treated and control mice (***p < 0.001).

A

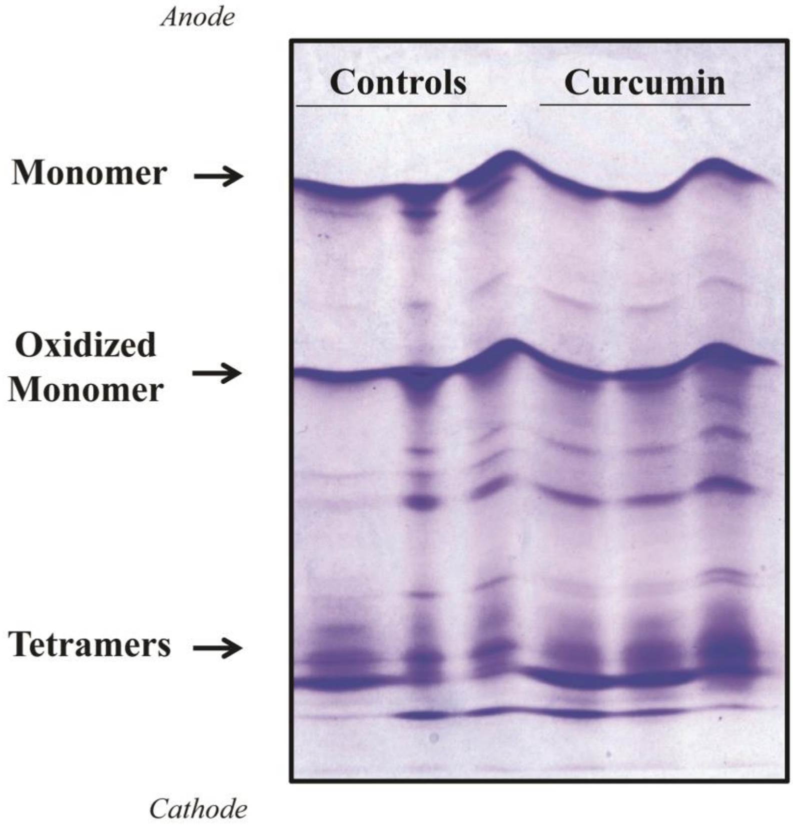

B

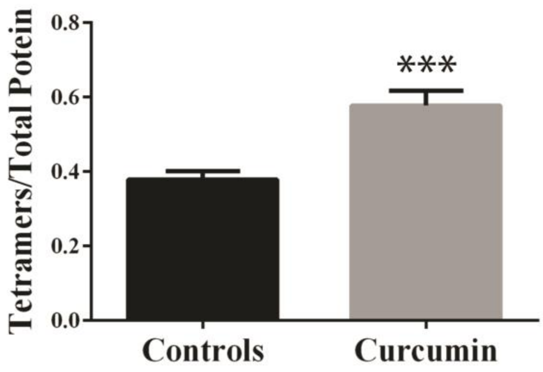

Supplement: Supplementary Information [file srep26623-s1.pdf]
